# Methotrexate in juvenile idiopathic arthritis: advice and recommendations from the MARAJIA expert consensus meeting

**DOI:** 10.1186/s12969-018-0255-8

**Published:** 2018-07-11

**Authors:** Giovanna Ferrara, Greta Mastrangelo, Patrizia Barone, Francesco La Torre, Silvana Martino, Giovanni Pappagallo, Angelo Ravelli, Andrea Taddio, Francesco Zulian, Rolando Cimaz, On behalf of the Rheumatology Italian Study Group

**Affiliations:** 1 University of Trieste, Trieste, Italy; 2 Rheumatology Unit, Anna Meyer Children Hospital and University of Florence, University of Florence, Florence, Italy; 3 Department of Pediatrics, University of Catania, Catania, Italy; 4 Pediatric Rheumatology Section, Pediatric Onco-Hematology Unit, Vito Fazzi Hospital, Lecce, Italy; 5 Clinica Pediatrica Università di Torino, Day-Hospital Immunoreumatologia, Turin, Italy; 6 Epidemiology & Clinical Trials Office, General Hospital, Mirano VE, Italy; 7 Pediatria II – Reumatologia, Istituto Giannina Gaslini, and Università degli Studi di Genova, Genoa, Italy; 8 Institute for Maternal and Child Health - IRCCS “Burlo Garofolo”, Trieste, and University of Trieste, Trieste, Italy; 9 Department of Pediatrics, Rheumatology Unit, University of Padua, Padua, Italy

**Keywords:** Juvenile idiopathic arthritis, Methotrexate, Consensus

## Abstract

**Background:**

Conventional pharmacological therapies for the treatment of juvenile idiopathic arthritis (JIA) consist of non-biological, disease-modifying antirheumatic drugs, among which methotrexate (MTX) is the most commonly prescribed. However, there is a lack of consensus-based clinical and therapeutic recommendations for the use of MTX in the management of patients with JIA. Therefore, the **M**ethotrexate **A**dvice and **R**ecommend**A**tions on **J**uvenile **I**diopathic **A**rthritis (MARAJIA) Expert Meeting was convened to develop evidence-based recommendations for the use of MTX in the treatment of JIA.

**Methods:**

The preliminary executive committee identified a total of 9 key clinical issues according to the population, intervention, comparator, outcome (PICO) approach, and performed an evidence-based, systematic, literature review. During the subsequent Expert Meeting, the relevant evidence was assessed and graded, and 10 recommendations were made.

**Results:**

Recommendations relating to the efficacy, optimal dosing and route of administration and duration of treatment with MTX in JIA, and to the issue of folic acid supplementation to prevent MTX side effects, use of MTX in the treatment of chronic JIA-associated uveitis, combination treatment with biologic agents, and the use of vaccinations in patients with JIA were developed. The selected topics were considered to represent clinically important issues facing clinicians caring for patients with JIA. Evidence was insufficient to formulate recommendations for the use of biomarkers predictive of treatment response.

**Conclusions:**

These consensus recommendations provide balanced and evidence-based recommendations designed to have broad value for physicians and healthcare clinicians involved in the clinical management of patients with JIA.

## Background

Juvenile idiopathic arthritis (JIA) is one of the most common chronic conditions of childhood. JIA comprises a group of heterogeneous forms of arthritis characteri**z**ed by persistent joint inflammation lasting longer than 6 weeks and beginning before the age of 16 years and has an unknown cause [1]. According to the classification criteria of the International League of Associations for Rheumatology (ILAR), the term JIA covers seven mutually exclusive categories with differences in their clinical presentation, disease course and treatment response, namely systemic arthritis, oligoarthritis, polyarthritis (rheumatoid factor negative), polyarthritis (rheumatoid factor positive), psoriatic arthritis, enthesitis-related arthritis, and undifferentiated arthritis [1]. Conventional pharmacological therapies consist of non-biological, disease-modifying antirheumatic drugs (DMARD), among which methotrexate (MTX) is the most commonly prescribed [2].

To date, despite the wide use of MTX, there is a lack of consensus-based clinical and therapeutic recommendations for the use of MTX in the management of patients with JIA. Only two papers, one recently-published article from the Spanish Society of Paediatric Rheumatology (Sociedad Española de Reumatología Pediátrica; SERPE) [3], and an older article by the Pediatric Immunology and Rheumatology Division of the Centre for Child Health, Heinrich-Heine-University, Düsseldorf, Germany [4] currently deal with this task.

Thus, the aim of our group was to develop evidence-based recommendations for the use of MTX in the treatment of juvenile idiopathic arthritis. To this end, the **M**ethotrexate **A**dvice and **R**ecommend**A**tions on **J**uvenile **I**diopathic **A**rthritis (MARAJIA) Expert Meeting was convened in Milan, Italy.

## Methodology

### Development of the research topics

Establishing recommendations requires the use of formal methods, such as the nominal group technique (NGT), which is based on discussions by an Expert Panel to gather opinions and define a degree of consensus for each statement.

A preliminary executive committee comprising Rolando Cimaz, Giovanna Ferrara and Greta Mastrangelo was responsible for identifying key clinical issues using the PICO (Population – Intervention – Comparator - Outcome) system [5], with the aim of: 1) defining research questions, and 2) developing criteria for selecting studies to be reviewed by the Expert Panel in the development of clinical and therapeutic recommendations for the management of MTX in patients with JIA. The PICO framework is designed to help researchers to achieve relevant and precise questions that can be answered in a systematic review structure, and allows improved specificity and conceptual clarity of the clinical question by splitting the questions into smaller manageable components which are more straightforward to identify in the literature search process.

The approach facilitates the identification of a precise definition of a group of participants (**Population**), clear reporting of the drug exposures (**Intervention**) and the control group interventions (**Comparator**) under consideration, and well-defined and clearly specified **Outcomes** of the intervention being assessed. Finally, the type of **Study design** to be included in the review should be reported.

The executive committee identified nine clinically important research topics relating to the use of MTX in JIA using a structured PICO process. The topics covered efficacy and safety, dosages, routes of administration, tapering, and discontinuation of MTX, folic acid supplementation, efficacy in JIA-associated uveitis, add-on therapy with biologic drugs, biomarkers, and vaccination. The selected topics were considered to represent clinically important issues facing clinicians caring for patients with JIA.

### Strategy for the literature search

A systematic search using PubMed and the Cochrane Library for human studies published in English until the present was conducted on the 30th of November 2016. The keywords used in the search were “juvenile idiopathic arthritis” and “methotrexate” (“arthritis, juvenile”[MeSH Terms] OR (“arthritis”[All Fields] AND “juvenile”[All Fields]) OR “juvenile arthritis”[All Fields] OR (“juvenile”[All Fields] AND “idiopathic”[All Fields] AND “arthritis”[All Fields]) OR “juvenile idiopathic arthritis”[All Fields]) AND (“methotrexate”[MeSH Terms] OR “methotrexate”[All Fields]).

### Study selection and data extraction

All papers found with the first search were initially selected as appropriate to the intended purpose on the basis of the title. Papers inconsistent with the main topic (for example for disease or drug) were excluded. A second revision and selection was made reading the abstracts of remaining papers. Then all studies identified were read in their full text.

### Critical appraisal of identified studies

Each of the included studies was assessed for level of evidence using Oxford criteria for evidence-based levels of evidence [6]. The levels of evidence used in the analyses are summarized in Table 1. Evidence levels are indicative of quality regarding confidence and study design. In defining the recommendations, the experts’ assessment of the clinical conclusions of the studies was combined with the definition of the evidence levels.

**Table 1:** Levels of evidence [6]

Levels of evidence	
1	Systematic review of all relevant randomized clinical trials or *n*-of-1 trials
2	Randomized trial or observational study with dramatic effect
3	Non-randomized controlled cohort/follow-up study (observational)
4	Case series, case-control study, or historically controlled study
5	Mechanism-based reasoning (expert opinion, based on physiology, animal or laboratory studies)
Grades of recommendation	
A	Consistent level 1 studies
B	Consistent level 2 or 3 studies, or extrapolations from level 1 studies
C	Level 4 studies, or extrapolations from level 2 or 3 studies
D	Level 5 evidence or troubling, inconsistent or inconclusive studies of any level

## Consensus process

### Expert panel composition

The Expert Panel participating in the MARAJIA Expert Consensus Meeting held in Milan, Italy on the 12th of April, 2017 to identify recommendations for the use of MTX in the treatment of JIA consisted of Patrizia Barone, Rolando Cimaz, Francesco La Torre, Silvana Martino, Angelo Ravelli, Andrea Taddio, and Francesco Zulian, under the methodological guidance of Giovanni Pappagallo. Giovanna Ferrara and Greta Mastrangelo were involved in formulating the PICO research topics and drafting the recommendation statements.

All experts were pediatric rheumatologists, the majority from tertiary centers with longstanding expertise in pediatric rheumatic diseases.

### Formulation of clinical recommendations

During the meeting, the Expert Panel considered the supporting research identified using the targeted literature search and formulated specific recommendation statements for each research topic. Ten clinical and therapeutic recommendations for the management of MTX were drafted and presented to the meeting with their supporting scientific evidence for discussion and voting by the Expert Panel towards reaching consensus.

The strength and relevance of the published evidence in support of a clinical intervention or treatment approach was evaluated, in addition taking into consideration the personal clinical experience of the panel participants. Each participant was required to express his or her expert opinion by rating the statement according to the following 7-point scale: 1) completely disagree; 2) somewhat disagree; 3) disagree a little; 4) neither agree nor disagree; 5) agree a little; 6) somewhat agree; 7) completely agree. A score of 6 or 7 was defined as “In favor”, 3, 4 or 5 as “Uncertain”, and 1 or 2 “Against”.

Through this process, all research statements achieved acceptance, with a second round of voting not required for any statement. One hundred percent agreement (a unanimous score of 7 on the 7-point scale) was obtained on 5 statements (Statements 2, 3, 4, 7 and 8) and 83% agreement on Statements 1 and 6 (7 or 6) and Statement 5 (7 or 6 with a single score of 5 from one Advisor). The research questions are detailed in Table 2.

**Table 2:** Summary of recommendations for the use of methotrexate in juvenile idiopathic arthritis

PICO research questions and recommendations	Grade of evidence	Supporting references
Research question 1: Efficacy and safety of methotrexate in juvenile idiopathic arthritis		
1. MTX is recommended as the first-line treatment in oligoarthritis that persists despite nonsteroidal anti-inflammatory drugs (NSAIDs) and intraarticular steroid (IAS) therapy, and in polyarticular disease	1A	[2–4, 7–15, 20, 21, 23–25]
MTX is also recommended in systemic arthritis with predominant joint inflammation, without active systemic features	4C	[2–4, 7–15, 20–25]
2. Clinical and laboratory monitoring of MTX toxicity is recommended every 4-8 weeks initially, and then every 12-16 weeks, unless risk factors are present	4C	[1, 4, 12, 21, 26–38, 40–42]
Research question 2: Dosages of methotrexate in juvenile idiopathic arthritis		
3. A dose of 10-15 mg/m^2^/week is recommended.	5D	[7, 9, 42]
Further increases in MTX dosage have not been associated with additional therapeutic benefit	1A	
Research question 3: Route of administration of methotrexate in juvenile idiopathic arthritis		
4. MTX may be given orally or subcutaneously once a week. If high doses (15 mg/m^2^/week) are requested, the subcutaneous route is preferable due to increased bioavailability	4C	[9, 21, 43–49]
Research question 4: Tapering and discontinuation of methotrexate in juvenile idiopathic arthritis		
5. MTX could be discontinued after 6 months of stable remission	1A	[50–52]
Research question 5: Folic acid supplementation for the prevention of methotrexate toxicity in patients with juvenile idiopathic arthritis		
6. Folic or folinic acid supplementation is recommended to prevent MTX side effects.	1A	[53–57, 59–62]
The advised dose is approximately one third of the MTX dose, at least 24 hours after the weekly dose of MTX for folinic acid; for folic acid 1 mg/day skipping the day when MTX is administered	4C	
Research question 6: Efficacy of methotrexate in uveitis associated with juvenile idiopathic arthritis		
7. MTX is recommended for the treatment of JIA-related uveitis refractory to topical treatment	4C	[63–72, 74–79]
Research question 7: Add-on therapy with biologic drugs in juvenile idiopathic arthritis not responding to methotrexate		
8. The combination of MTX with a TNF-α inhibitor is recommended in patients who had an inadequate clinical response to MTX alone	3B	[11, 48, 80, 83–85, 88, 89]
Combination therapy is safe and may reduce the development of anti-drug antibodies	2B	[83, 88–90]
Research question 8: Molecular elements and genetic markers of response to methotrexate in juvenile idiopathic arthritis – Biomarkers		
9. No recommendation is made regarding the use of biomarkers in current clinical practice		[91–101]
Research question 9: Use of vaccination in patients with juvenile idiopathic arthritis treated with methotrexate		
10. Vaccination with non-live vaccines is not contraindicated during MTX treatment	2B	[101–119]
No recommendation can be formulated for live-attenuated vaccines, but the available data for measles, mumps, rubella (MMR) booster indicate that it is safe and adequately immunogenic		

Abbreviations: *IAS* intra-articular steroid, *JIA* juvenile idiopathic arthritis, *MMR* measles, mumps, rubella, *MTX* methotrexate, *NSAIDs* nonsteroidal anti-inflammatory drugs, *TNF-α* tumor necrosis factor-α

## Research strategy and evidence selection

We obtained 843 references in our literature search. Among these, we selected 209 relevant references, of which 33 were clinical trials, 51 reviews, 1 Cochrane meta-analysis and 124 articles of other types.

**Fig. 1:**
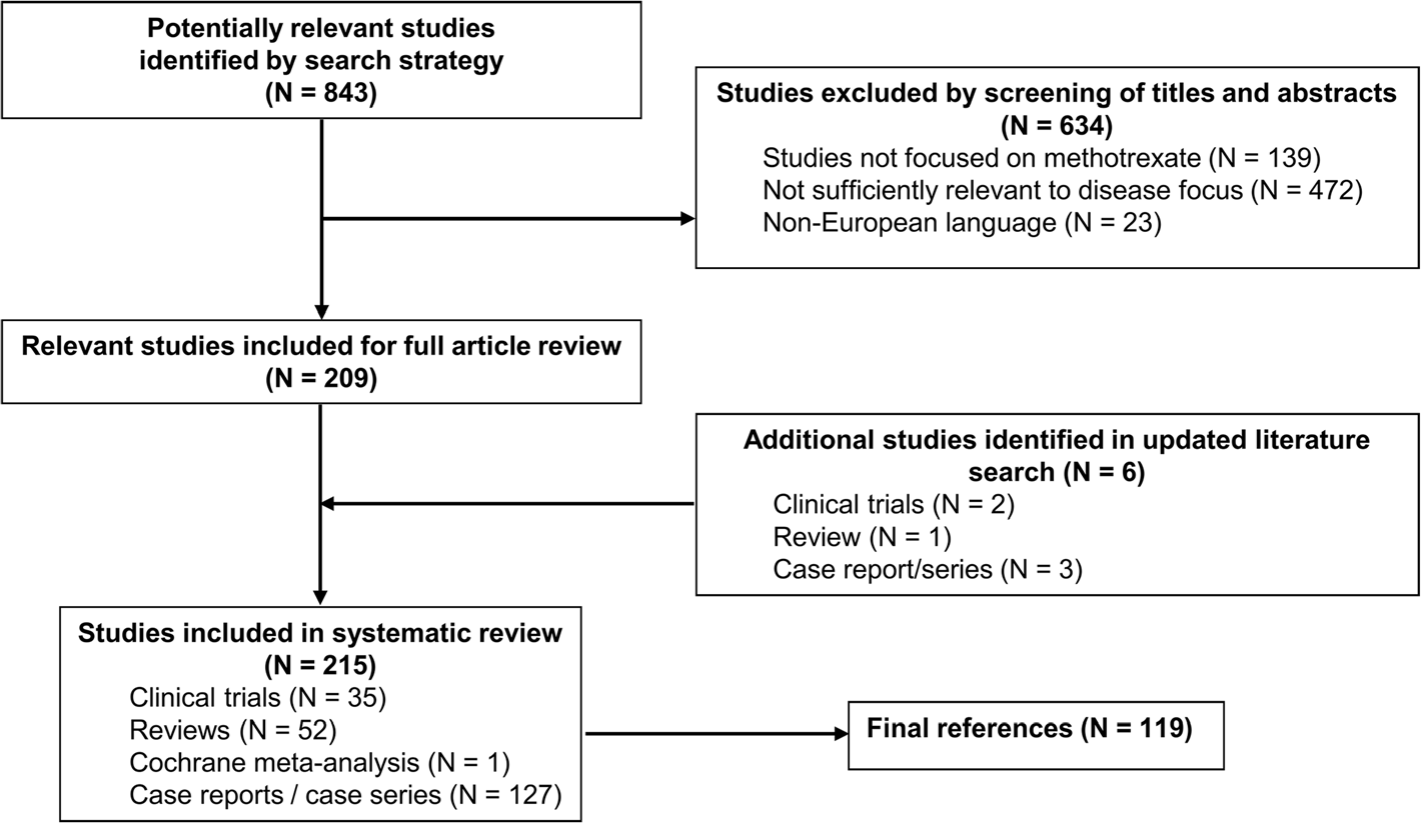
Study selection process flow diagram

A total of 472 references were excluded because they were judged not to be relevant, 139 because the studies were mainly about biologic drugs and there was an insufficient focus on MTX, and 23 because they were published in non-European languages. Six articles (2 clinical trials, 1 review, and 3 articles of other types) were subsequently included from an updated literature search (28 February 2017). A flow diagram of the study selection process is shown in Fig. 1.

## Methotrexate in juvenile idiopathic arthritis: Recommendations for use

A summary of the recommendations for the use of MTX in JIA for each of the PICO research questions is presented in Table 2.

### Research question 1: Efficacy and safety of methotrexate in juvenile idiopathic arthritis

**Recommendation 1.** *MTX is recommended as the first-line treatment in oligoarthritis that persists despite nonsteroidal anti-inflammatory drugs (NSAIDs) and intraarticular steroid (IAS) therapy, and in polyarticular disease (Evidence Grade 1A).*

*MTX is also recommended in systemic arthritis with predominant joint inflammation, without active systemic features (Evidence Grade 4C)*.

**Recommendation 2.** *Clinical and laboratory monitoring of MTX toxicity is recommended every 4–8 weeks initially, and then every 12–16 weeks, unless risk factors are present (Evidence Grade 4C).*

PICO framework: P: children affected by JIA; I: administration of MTX; C: placebo or other therapies (salazopyrin, oral steroids, NSAIDs); O: efficacy and safety.

MTX is the most widely used DMARD in the treatment of JIA. A folic acid analog and an inhibitor of several different enzymes in the folate pathway, MTX exerts immunomodulatory and anti-inflammatory actions. Its efficacy was first demonstrated in a randomized controlled trial more than two decades ago [7]. MTX has been studied in further controlled clinical trials [8, 9] and has been established as the most common first-line DMARD treatment according to several national treatment guidelines [10–13]. In particular, considering the categories of JIA, NSAIDs and IAS therapy remain the first choice in oligoarthritis [1]. Furthermore, a recent multicenter, prospective, randomized, open-label trial [14] found that concomitant administration of MTX did not augment the effectiveness of intra-articular corticosteroid therapy.

MTX is recommended as first-line treatment in polyarthritis, and in systemic arthritis with predominant joint inflammation [2, 8, 11, 15]. However, initiation of sulfasalazine (SSZ) is recommended following IAS or an adequate trial of NSAIDs for patients with the enthesitis-related arthritis category of JIA, with moderate activity [11]. Sulfasalazine has never been compared with MTX in treating JIA.

Currently, there are no published recommendations for the treatment of juvenile spondyloarthropathies. The ACR recommendations for the management of JIA suggest the use of sulfasalazine for patients with enthesitis-related arthritis. This recommendation is based on clinical experience and data from adult patients with ankylosing spondylitis. However, in the adult population it has been shown that sulfasalazine is ineffective in axial disease, while several observational studies have found that tumor necrosis factor (TNF)-α inhibitors are beneficial in juvenile spondyloarthropathies [16–19]. Furthermore, a recent randomized controlled trial demonstrated the efficacy of adalimumab in enthesitis-related arthritis [16, 18]. Available studies suggest that TNF-α inhibitors should be used when sulfasalazine is ineffective or earlier in moderate or highly active axial disease with established radiographic damage, such as erosions or joint-space narrowing.

MTX has been shown to be an effective drug in the indication, with 65–90% of patients successfully responding to treatment [9, 20–22]. MTX also significantly improved a wide range of health-related quality-of-life components, particularly in the physical domains [23].

Despite what has previously been reported in adult patients, MTX may also slow the radiologic progression of disease in JIA, acting as a disease-modifying drug, although the studies available involved few children [24, 25].

During 30 years of its use, MTX has shown a good safety profile, with few severe side effects reported [26]. Nevertheless, more than half of children were reported to have difficulties in taking it [27, 28]. The most common side effect of MTX include nausea or vomiting and abnormalities in liver function tests, the latest reported in 10–20% of patients [29, 30]. However, the transaminase levels usually normalize one or two weeks after stopping therapy. Others symptoms are mouth sores, rash, diarrhea and laboratory abnormalities such as leukopenia and hypogammaglobulinemia that may predispose to infections. Alopecia is seen in some patients, but hair grows back after stopping the medication. Since photosensitivity has been reported, limiting sun exposure and the use of sunscreen is advised. It is worth remembering that MTX is teratogenic, and it is necessary to use contraception while taking the drug and for 3–6 months after discontinuation [29].

MTX may cause cirrhosis and lung fibrosis, but these are extremely rare and have been reported only in adults with other comorbidities [31–33]. In the literature there are also few reports of lymphoma in children treated with MTX [34–36], but it has not been possible to determine whether these observations were merely coincidental, were causally linked to MTX, or were related to the underlying disease process. The issue of whether MTX treatment is an independent risk factor for various malignancies is controversial and remains unresolved. Long-term prospective cohort studies are needed to define the risk of hematological or other malignancies in MTX-treated patients.

Nodulosis is a rare MTX adverse event that has been described in JIA (accelerated nodulosis in two teenagers with rheumatoid factor [RF]-positive juvenile rheumatoid arthritis and one 3-year-old girl with systemic-onset disease). The nodules developed within six months after the initiation of MTX treatment and regressed after discontinuing therapy, or were successfully treated with hydroxychloroquine or colchicine [37–39].

Regarding laboratory monitoring in patients with JIA, there is only one guideline by Ortiz-Alvarez et al., derived from the American College of Rheumatology (ACR) guidelines for monitoring MTX toxicity in adults. They suggest a complete and differential blood count, liver function tests and albumin and serum creatinine levels every 4–8 week initially, and then every 12–16 week, unless risk factors are present [40]. Other authors also recommended hepatitis and varicella-zoster virus serology and tuberculin test before starting therapy [4]. MTX is contraindicated in children with reduced renal function.

Bulatović et al. [27] designed and validated the MTX Intolerance Severity Score (MISS) questionnaire to identify patients with MTX intolerance. The items investigated were: abdominal signs and symptoms (pain, nausea, vomiting) and behavioral symptoms (restless, crying, irritability, and refusal of MTX) before and after the administration of MTX. A cut-off score of 6 yielded the best sensitivity (88%) and specificity (80%). They found there was no difference in efficacy between the various routes of MTX administration. However, half of the 297 patients were MTX-intolerant. This was especially the case in patients who received parenteral MTX, who experienced more anticipatory behavioral symptoms prior to administration, compared to patients receiving oral MTX. However, the difference in the prevalence of gastrointestinal symptoms was not great.

Van Dijkhuizen et al. [41] also found more side effects among patients who received parenteral MTX. On the other hand, Klein et al. [21] found no difference in the prevalence of side effects between oral and parenteral MTX.

Overall, analysis of available studies and clinical experience of the participating experts show that MTX is usually well tolerated in patients with JIA.

### Research question 2: Dosages of methotrexate in juvenile idiopathic arthritis

**Recommendation 3.** *A dose of 10–15 mg/m* ^*2*^ */week is recommended (Evidence Grade 5D). Further increases in MTX dosage have not been associated with additional therapeutic benefit (Evidence Grade 1A).*

PICO framework: P: children affected by JIA on treatment with MTX; I: low dosage of MTX (< 10 mg/m^2^/week); C: high dosage of MTX (> 10 mg/m^2^/week); O: efficacy and safety.

The therapeutic range of MTX for JIA is 8.5–15 mg/m^2^/week. The first study by Giannini et al. showed that a dose of 5 mg/m^2^/week was not superior to placebo, while 15 mg/m^2^/week was superior to 10 mg/m^2^/week [7].

Children seem to tolerate much higher doses than adults, and some series have described using 20 to 25 mg/m^2^/week or 1.1 mg/kg/week in children with resistant disease with relative safety in the short-term [42]. However, a multinational, randomized controlled study confirmed this therapeutic range and showed no benefit of doses above 15 mg/m^2^/week [9].

### Research question 3: Route of administration of methotrexate in juvenile idiopathic arthritis

**Recommendation 4.** *MTX may be given orally or subcutaneously once a week. If high doses (15 mg/m*^*2*^*/week) are requested, the subcutaneous route is preferable due to increased bioavailability* (*Evidence Grade 4C).*

PICO framework: P: children affected by JIA on treatment with MTX; I: subcutaneous administration of MTX; C: oral administration of MTX; O: efficacy, safety, and tolerability.

There is significant intraindividual and interindividual variability in the absorption and pharmacokinetics of MTX after oral administration [43, 44].

A pharmacokinetic study showed that factors such as age, body weight, creatinine clearance, gender, dose, and fasting state significantly influenced the absorption of MTX in adults with rheumatoid arthritis. The bioavailability of MTX has also been shown to be greater in the fasting state in children with JIA [45].

MTX should be taken on an empty stomach with water or clear beverages. Oral bioavailability generally is about 15% less than after intramuscular administration. The bioavailability of intramuscular and subcutaneous administration is similar, with the latter being generally more acceptable for children who require parenteral MTX [46, 47].

Several studies have reported the successful use of parenteral treatment in non-responders to oral MTX treatment, but there are no controlled comparative studies (only open-label studies are available). Alsufyani et al. found that patients switching from oral to subcutaneous administration of MTX had a 70% improvement in response [48].

Klein et al. in a retrospective study showed no differences in effectiveness between oral and parenteral administration of MTX, even if more patients on parenteral therapy discontinued it [21]. In clinical practice, MTX is preferentially administrated subcutaneously, and there is no sound study demonstrating greater efficacy for the oral route of MTX administration.

At doses over 15 mg/m^2^/week, the parenteral route may be better because of the decreased oral bioavailability of the drug at high doses. It has been shown that subcutaneous administration of MTX has a 10–12% increased absorption compared with oral preparations [46, 49]. In discussion amongst the Panel members, it was noted that in clinical practice, treatment is usually started with MTX 15 mg/m^2^/week, particularly in severe forms of JIA, where the patient is directly treated with MTX 15 mg/m^2^/week. The Panel further suggested that, for the first administrations, the starting dose can be 10 mg/m^2^/week, and then the dose can be increased at subsequent MTX administrations, if necessary. Ruperto et al. reported that MTX doses greater than 15 mg/m^2^/week provided no additional clinical benefit, and that this dose should not be exceeded [9].

### Research question 4: Tapering and discontinuation of methotrexate in juvenile idiopathic arthritis

**Recommendation 5.** *MTX could be discontinued after 6 months of stable remission (Evidence Grade 1A).*

PICO framework: P: children affected by JIA on treatment with MTX; I: tapering and discontinuing treatment six months after achieving remission; C: discontinuing MTX twelve months or longer after achieving remission; O: survival free of flares after stopping treatment.

MTX is a slow-acting drug, generally displaying its full therapeutic effect in 6–8 weeks (from 3 to 18 weeks among different studies), so there is general agreement to wait at least 12 weeks to assess its efficacy. On the contrary, there is a wide variability on the tapering and discontinuation of MTX doses in everyday clinical practice. There have been many studies in children treated with variable doses of MTX for variable periods in whom discontinuation of MTX was attempted after clinical “remission” of variable length was achieved [50].

The criteria for “remission” or “relapse” have usually not been well defined or standardized among various studies, and the assessment of outcomes has been nonblinded. Only Foell et al. in a randomized clinical trial proved the safety of withdrawing MTX therapy after 6 months of stable remission versus 12 months. The results of this study that included 364 patients showed that a 12-month versus 6-month withdrawal of MTX did not reduce the relapse rate [51].

MTX withdrawal may result in disease flare in more than 50% of patients, and even more in younger children. A longer period on MTX treatment after remission may not prolong the duration of improvement after stopping treatment, but the duration of clinical remission may be predicted by the degree of subclinical synovial inflammation (using myeloid related proteins 8 and 14 [MRP8/MRP14]) at the time of stopping MTX [52].

### Research question 5: Folic acid supplementation for the prevention of methotrexate toxicity in patients with juvenile idiopathic arthritis

**Recommendation 6.** *Folic or folinic acid supplementation is recommended to prevent MTX side effects (Evidence Grade 1A). The advised dose is approximately one third of the MTX dose, at least 24 h after the weekly dose of MTX for folinic acid; for folic acid 1 mg/day skipping the day when MTX is administered (Evidence Grade 4C).*

PICO framework: P: children affected by JIA on treatment with MTX; I: MTX and folic acid supplementation; C: MTX alone; O: frequency (prevalence/incidence) of nausea and dyspepsia.

MTX toxicity, such as hepatotoxicity, hematologic changes, gastrointestinal and mucocutaneous intolerance, has been hypothesized to be a result of an induced state of folate depletion. The addition of folate, therefore, can counteract the signs of toxicity, either as folic or folinic acid (a reduced form of folic acid), since they can function in biosynthetic pathways independent of dihydrofolate reductase.

In a double-blind placebo-controlled study in RA, 1–5 mg of folic acid led to a significant reduction of side effects whilst preserving the efficacy of MTX therapy, even if, in order to preserve the anti-inflammatory effect, a slightly higher dosage of MTX was necessary [53]. Several clinical studies showed also that folic acid supplementation is associated with a reduced MTX discontinuation rate [53–55]. According to available data, folic acid supplementation does not appear to interfere with the therapeutic efficacy of MTX [55–57]. Indeed, there is increasing evidence that the anti-inflammatory effect of MTX is mediated by adenosine and is unrelated to folic or folinic acid [58]. A randomized controlled study, which directly compared folic acid to folinic acid in rheumatoid arthritis, showed no difference between the two forms of supplementation [53].

Studies in children are limited. A 13-week, randomized, double-blind, placebo-controlled, crossover trial of folic acid (1 mg/day) or placebo combined with a stable dose of MTX in 19 children with juvenile rheumatoid arthritis reported no effect on the clinical efficacy of oral weekly MTX. No liver function tests abnormalities were observed, but no data about other toxicities were available [59]. According to the findings of the studies conducted in adults, the frequency of increased transaminases is reduced by 60% by folinic acid supplementation [57]. Furthermore, in a retrospective non-controlled study [60] the efficacy of folinic acid supplementation was investigated in a cohort of 43 children on an intermediate dose of MTX. A significant reduction in hepatotoxicity and gastrointestinal toxicity was shown, without compromising MTX efficacy.

Administration of folic or folinic acid 24 h apart from the administration of MTX, in a dose of approximately one-third of the MTX dose, has been used to prevent MTX toxicity manifestations [61].

However, in limited cases, it is reported that at high doses folic acid supplementation seems to be associated with disease flares [62].

According to available data, it is not possible to make firm recommendations about routine folate supplementation in children receiving MTX treatment. However, data from adult studies and limited pediatric data can provide helpful information. Low-dose (1 mg/day) folic acid supplementation does not affect the anti-inflammatory efficacy of MTX and counteracts the signs of gastrointestinal and mucosal toxicities associated with it. The advisable dose is approximately one-third of the MTX dose, at least 24 h after the weekly dose of MTX, or 1 mg/day skipping the day when MTX is administered (Grade 4C). Folic acid supplementation does not appear to interfere with the therapeutic efficacy of MTX and seems to be associated with a reduced MTX discontinuation rate.

### Research question 6: Efficacy of methotrexate in uveitis associated with juvenile idiopathic arthritis

**Recommendation 7.** *MTX is recommended for the treatment of JIA-related uveitis refractory to topical treatment (Evidence Grade 4C).*

PICO framework: P: children affected by JIA and uveitis; I: administration of MTX; C: placebo or other therapies (e.g., oral steroids); O: efficacy and safety.

Although there is a lack of randomized controlled studies on the subject, the available data suggest that MTX is useful for preventing the onset of uveitis and improving disease activity in cases of JIA. In particular, a systematic review and meta-analysis of prospective studies carried out by Simonini et al. found that there was a 73% (95% confidence interval 66–81%) likelihood of improving intraocular inflammation in patients treated with MTX [63]. The systematic review was based on data from nine retrospective chart reviews [63–72]. The number of children in studies varied from 3 to 25, and the dose of MTX ranged from 7.5 to 30 mg/m^2^, with 15 mg/m^2^ the most commonly used. Ninety-five of 135 children were responders to MTX. The outcome measures to assess the effectiveness of MTX were collected according to the Standardization of Uveitis Nomenclature working group criteria [73]. It was reported that additional topical steroids or systemic immunosuppressive drugs were often required. However, the lack of randomized controlled trials means that treatment with immunosuppressive drugs is supported only at evidence level III: expert opinion, clinical experience or descriptive studies [74].

Additionally, Charuvanij and colleagues [75] reviewed the medication history in 43 children with JIA and anterior uveitis. Topical corticosteroids alone permitted satisfactory disease control in few patients (16%). The addition of MTX controlled the uveitis in three-quarters of patients, but additional systemic immunosuppressive drug (infliximab) was required in 6 children, with disease control in 4 patients.

The lack of evidence from randomized controlled trials limits our understanding of MTX effectiveness in the indication and of the best time to start therapy, even though MTX is largely used in chronic uveitis, mostly when associated with JIA.

Heiligenhaus et al. [76] suggest adding an immunosoppressive drug (i.e. MTX) to steroids when the inflammation in the eyes has not resolved within 12 weeks under treatment with topical corticosteroids maximally 3 times daily or, in cases of recurring uveitis, under a systemic corticosteroid dosage of more than 0.15 mg/kg body weight or if new uveitis complications develop. The preferred dose is 15 mg/m^2^/week (maximum 25 mg/m^2^/week) [77]. Evidence from several sources suggested that if MTX is effective in controlling inflammation, treatment should be maintained for 12 months from when inactive uveitis has been confirmed. In patients with poor visual prognosis, MTX treatment should be maintained over 24 months [77].

In terms of preventing the onset of uveitis in children during early treatment with MTX Papadopoulou et al. [78] performed a retrospective study of 254 patients with JIA. Eighty-six patients (33.9%) were treated with MTX and 168 patients (66.1%) did not receive MTX. Over the 2-year follow-up, the frequency of uveitis was lower in patients who had received MTX than in untreated patients (10.5% vs 20.2%, respectively, *p* = 0.049). The majority of patients in the study had persistent and extended oligoarthritis (61.8 and 22.4%, respectively); 14.2% of patients had RF-negative polyarthritis. As expected, patients treated with MTX had a greater frequency of polyarticular disease, which is well known to have a lower incidence of uveitis. However, the distribution of the main risk factors for uveitis (proportion of female and antinuclear antibodies- positive subjects) and the median age at disease onset were comparable between the two groups. In a longitudinal analysis from a nationwide pediatric rheumatology database [79] the influence of MTX, TNF-α inhibitors, and a combination of the 2 medications on uveitis occurrence in JIA patients was analyzed. In a total of 3512 patients the use of any of these drugs in the year before uveitis onset significantly reduced the risk for uveitis, and the use of MTX within the first year of disease and of the combination of MTX with a TNF-α inhibitor had the highest protective effect.

In a recent systematic review [63], MTX seems an effective therapy for uveitis associated with JIA.

### Research question 7: Add-on therapy with biologic drugs in patients with juvenile idiopathic arthritis not responding to methotrexate

**Recommendation 8.** *The combination of MTX with a TNF-α inhibitor is recommended in patients who had an inadequate clinical response to MTX alone (Evidence Grade 3B). Combination therapy is safe and may reduce the development of anti-drug antibodies (Evidence Grade 2B).*

PICO framework: P: children affected by JIA on treatment with MTX who did not achieve remission; I: MTX plus TNF-α inhibitors (etanercept and adalimumab); C: MTX alone; O: efficacy and safety.

The ACR recommendations [11] propose the addition of a TNF-α inhibitor (etanercept or adalimumab) for patients who had a partial previous clinical response to MTX with persistent disease activity, recommending, after starting combination therapy, that treatment with MTX be continued or not depending on the patient’s previous response to it.

Two retrospective cohort studies recommended completion of a maximal response timeframe and to achieve the maximum effective dose by the parenteral route before considering combination therapy [48, 80].

Studies in adult patients with RA revealed a superiority of combining MTX and etanercept versus MTX only [81, 82]. This combination has also been successfully used in children and adolescents, despite a lack of double-blind, randomized controlled trials [83].

A 3-year, open-label, prospective multicenter study of children and adolescents (aged 2–18 years) with polyarticular, systemic, or extended oligoarticular JIA receiving MTX (*n* = 197), etanercept (*n* = 103), or both (*n* = 294) showed good safety and efficacy in all three groups. The results of this study indicated that patients with polyarticular (RF-positive or negative) or systemic JIA benefit from etanercept or etanercept plus MTX treatment [84]. Improvements in joint counts and physician’s global assessment scores were similar across three different arms, and improvements were maintained for three years in those continuing to receive medication.

In a randomized, double-blind, stratified, placebo-controlled, multicenter, medication-withdrawal study with a 16-week open-label lead-in phase, a 32-week double-blind withdrawal phase, and an open-label extension phase, 171 children with active juvenile rheumatoid arthritis underwent stratification according to MTX use (85 patients receiving MTX, 86 not receiving MTX) and received adalimumab every other week for 16 weeks. Subsequently, those that had an ACR Pediatric 30% (ACR Pedi 30) response at week 16 (74% of patients not receiving MTX and 94% of those receiving MTX) were randomly assigned to receive adalimumab or placebo in a double-blind fashion every other week for up to 32 weeks. At 48 weeks, the percentages of patients treated with MTX who had ACR Pedi 30, 50, 70, or 90 responses were significantly greater for those receiving adalimumab than for those receiving placebo; the differences between patients not treated with MTX who received adalimumab and those who received placebo were not significant. The study was not statistically powered to detect differences between patients receiving and those not receiving MTX; however, the proportions of patients with ACR Pedi 30, 50, 70, or 90 responses were somewhat higher among those receiving adalimumab in combination with MTX than among those receiving adalimumab without MTX [85].

A diminished response to treatment with certain TNF-α inhibitors may be associated with the development of anti-drug antibodies [86], and concomitant use of MTX reduces the immunogenicity of these drugs [85, 87].

Concerning safety, several studies reported that MTX combined with anti-TNF-α does not increase its toxicity [83, 88–90].

MTX in combination with biologic therapy is safe and may reduce the development of anti-drug antibodies in addition to improving response.

### Research question 8: Molecular elements and genetic markers of response to methotrexate in juvenile idiopathic arthritis – Biomarkers

**Recommendation 9.** *No recommendation is made regarding the use of biomarkers in current clinical practice.*

PICO framework: P: children affected by JIA undergoing treatment with MTX; I: evaluating the concentration of MTX polyglutamates and genetics variants in MTX responders; C: polyglutamate levels and genetic variants in patients with JIA non-responders to MTX; O: response to MTX in children with JIA.

Although MTX is the first choice in JIA, it is known that about one-third of patients fail to respond. Given the time lag between MTX treatment initiation and the patient response (about 3 months), it would be particularly useful to determine a priori the probability of beneficial therapeutic response [91, 92].

In fact, the delay in identifying the optimal treatment at an early stage of disease can influence long-term joint damage. Several biomarkers have been investigated so far. Recent studies found that the effect of MTX in JIA is associated with MTX polyglutamate intracellular concentrations: elevated long chain MTX polyglutamate levels are associated with lower disease activity indexes (JADAS) during 1 year of MTX treatment in JIA [93–95].

Other studies have evaluated the effects of genetic variants in the complex pathway of candidate genes involved in MTX pharmacokinetics and pharmacodynamics on the response to the medication in children with JIA. These studies found that genetic variants that predict MTX response in JIA are those in 5-aminoimidazole-4-carboxamide ribonucleotide-transformylase (ATIC), inosine triphosphate-pyrophosphatase (ITPA) and SLC19A1 genes [96–99].

Pastore et al. showed that reduced activity of ITPA, an enzyme involved in nucleotides’ homeostasis, is related to reduced MTX efficacy in patients with JIA [100]. The same group also found that a common functional variant in ATIC gene is associated with good response to MTX, while a variant in ITPA is associated with reduced response to MTX. However, there are suggestions that genetic variability, specifically single-nucleotide polymorphisms (SNP), in MTX metabolic pathways may be a better marker for MTX toxicity than for efficacy [99].

The conclusions of these studies may suggest that patients with variants associated with lack of efficacy for MTX should be switched more rapidly to a more aggressive treatment, but studies specifically addressing this issue are still lacking. In the future, therapy personalization in JIA may be achieved by a pharmacological approach integrating pharmacokinetic and pharmacogenomic evaluations. However, the supporting evidence is not yet sufficiently robust to form the basis of a recommendation.

Therefore, it was determined that the place of pharmacokinetic and pharmacogenomic analysis performed before MTX treatment in patients with JIA to identify those predisposed to better responses currently undefined and, furthermore, in current clinical practice no assessment of the biomarkers predictive of treatment response is carried out. Therefore, it was decided that no recommendation regarding the use of biomarkers in the treatment of patients with JIA should be made.

### Research question 9: Use of vaccination in patients with juvenile idiopathic arthritis treated with methotrexate

**Recommendation 10.** *Vaccination with non-live vaccines is not contraindicated during MTX treatment (Evidence Grade 2B).*

*No recommendation can be formulated for live-attenuated vaccines, but the available data for measles, mumps, rubella (MMR) booster indicate that it is safe and adequately immunogenic.*

PICO framework: P: children affected by JIA on treatment with MTX; I: vaccinations during treatment with MTX; C: no vaccinations during treatment with MTX; O: safety and efficacy of vaccinations, safety of drugs.

Considering that children with JIA have an increased risk of infection, which contributes to the morbidity of their disease, non-live vaccines, and live-attenuated vaccines can be recommended in these patients. However, the presence of immunosuppressive drugs can interfere with effectiveness and safety of vaccinations.

In 2011, the European League Against Rheumatism (EULAR) published recommendations regarding the vaccination of children with rheumatic diseases [101], based on a systematic literature review published in that same year [102]. The EULAR guidelines recommend adherence to the national vaccination guidelines for live-attenuated vaccines in pediatric patients unless the patients are on high-dose immunosuppressants, high-dose cortisone or biological agents. Booster vaccinations against varicella, yellow fever, and measles, mumps, rubella (MMR) can be considered in patients receiving MTX less than15mg/m^2^ or low-dose corticosteroids. However, it should be noted that the MTX summary of product characteristics states that live vaccines are contraindicated in patients taking MTX.

Recently Groot et colleagues provided an update to July 2014 of the systematic literature study of 2011 [103]. Eight studies on MTX and vaccinations, counting in total 420 patients, were available in the Groot review concerning the most common vaccines, i.e., seasonal influenza virus and H1N1, hepatitis B virus (HBV), meningococcus C, pneumococcus, measles, mumps and rubella (MMR), varicella zoster virus (VZV), bacillus Calmette-Guérin (BCG) [104–111]. Further we found five more articles about the above-mentioned vaccines including subgroups of patients on MTX [112–116], and one more concerning the bivalent human papillomavirus (HPV) vaccine [117].

In a prospective controlled observational cohort study, the immunogenicity of the bivalent HPV 16/18 vaccine in 68 patients with JIA was compared to 55 healthy controls, showing that all participants were seropositive up to 12 months after vaccination. No deleterious effect of MTX on antibodies was detected in the subgroup of 24 patients on MTX. No relevant differences in adverse events were found, and HPV vaccination did not aggravate JIA disease activity [117].

In two prospective open-label studies, influenza vaccine response and safety among patients treated with MTX were compared with a control group. Both studies showed that influenza vaccination in JIA induces a lower but effective protective antibody response with an adequate disease safety profile [109, 111].

Kasapcopur et al. compared responsiveness and safety of hepatitis B vaccination in 39 children affected by JIA and 41 healthy children. No effect of MTX on antibody concentration or response rate and no increase in disease activity were observed. A vaccination schedule at 0, 1, 6 months appeared to be the most effective [105].

The Neisseria meningitidis C (NeisVac-C) vaccine, is also safe and immunogenic in patients with JIA [115]. A retrospective cohort study showed that persistence of MenC-specific immunoglobulin (Ig)G antibodies in patients with JIA is similar to healthy controls and there is no effect of MTX on the decline of antibody levels over time, unlike biologicals [107].

Farmaki and colleagues observed that patients with JIA, when using MTX, had a similar response and seroprotection rate to the 7-valent pneumococcal vaccine (PCV7) as in healthy controls [114]. The only study evaluating the 23-valent polysaccharide pneumococcal vaccine in patients with JIA also demonstrated vaccine safety and effectiveness [116].

In a randomized, multicenter, open-label clinical equivalence trial, 137 patients with JIA aged 4 to 9 years (60 using MTX and 15 using biologics) were randomly assigned to receive MMR booster vaccination (*n* = 68) or no vaccination (control group; *n* = 69). Disease activity during complete follow-up did not differ between revaccinated patients and controls and seroprotection rates at 12 months after vaccination were higher in revaccinated patients. It seems that MTX and biologics did not affect humoral responses, but low patient numbers precluded definite conclusions. Moreover, no disease due to infections with attenuated viruses occurred in patients treated with immunosuppressive drugs [113]. A retrospective cross-sectional study [104], a retrospective observational multicenter cohort study [108] and a prospective case-control study [110] confirm these results.

In a prospective study [112], safety and immunogenicity of the VZV vaccine among 25 patients with pediatric rheumatic diseases treated with MTX and corticosteroids were compared to 18 healthy children. The vaccine proved to be safe in MTX treated patients, and no severe treatment-related adverse effects were observed during the one year follow up period. Both patients and controls had a low seroconversion rate one year after vaccination. However, a recent study [118] showed a low seroconversion rate in susceptible healthy children after one dose of vaccine, and indeed the US Centers for Disease Control and Prevention (CDC) guidelines advocate the use of 2 doses [119]. In clinical practice, a booster dose of VZV vaccine is normally administered to patients who fail to exhibit an immunological response after the first dose.

Kiray et al. demonstrated that there is no effect of MTX on purified protein derivative (PPD) induration size several years after BCG vaccination [106]. The PPD positivity rate was similar in MTX users and nonusers, even if the response to PPD was significantly lower in BCG-vaccinated children with JIA compared to healthy children. However, because of the lack of safety data, BCG vaccinations should not be administered to patients on immunosuppressive drugs (including MTX) or biologicals.

According to available data, no detrimental effect of MTX on the short-term immunogenicity or on the persistence of antibodies over time and no relevant increase in vaccine-associated adverse events were found in patients treated with MTX. Non-live vaccines are generally adequately immunogenic and safe. It appears that live-attenuated vaccines can be safely and effectively administered to patients with JIA on MTX, unless they are also on additional immunosuppressive drugs or biologicals. In these cases, evidence on safety is scarce. Live-attenuated booster vaccinations can be considered on an individual basis, although the data do not currently support the formulation of a specific recommendation for live-attenuated vaccines. There is no evidence in pediatric patients about the safe time intervals for the administration of live vaccines after cessation of immunosuppressive/immunomodulatory drugs such as MTX.

## Discussion

Although MTX is accepted as the most effective non-biologic agent for the treatment of patients with JIA, there is a wide variability in everyday practice in the use of MTX in the management of JIA. Therefore, the adoption of a consensus approach by a group of practitioners expert in the use of the drug in treating patients with JIA has the potential to guide clinicians and improve the understanding and management of this condition.

The recommendations presented in these consensus guidelines developed by the panel of experts participating in the MARAJIA Expert Consensus Meeting are based on a high level of evidence provided in large measure by robust data from randomized controlled clinical trials. Based on a set of key clinical issues developed using the PICO system and a rigorous approach to evidence review and the formulation of the research questions adopted to reduce the introduction of biases and to ensure balanced and evidence-based recommendations, we identified sound scientific support to guide the use of MTX in patients with JIA. Our consensus-based analysis integrated the scientific evidence from the literature with clinical experience to provide a set of recommendations we believe are of value in helping clinicians optimize the treatment of their patients with a diagnosis of JIA.

## Conclusions

These consensus recommendations relating to the efficacy, optimal dosing and route of administration and duration of treatment with MTX in JIA, and to the important issues of folic acid supplementation to prevent MTX side effects, use of MTX in the treatment of chronic JIA-associated uveitis, combination treatment with biologic agents, and the use of vaccinations in patients with JIA provide balanced and evidence-based recommendations designed to have broad value for physicians and healthcare clinicians. We did not at this time find sufficient evidence to justify pharmacokinetic and pharmacogenomic analysis prior to MTX treatment in current clinical practice, as insufficient evidence is available on biomarkers able to predict treatment response.

## Abbreviations

ACR: American College of Rheumatology
BCG: Bacillus Calmette- Guérin
DMARD: Disease-modifying antirheumatic drugs
EULAR: European League Against Rheumatism
HBV: Hepatitis B virus
HPV: Human papillomavirus
IAS: Intraarticular steroid
Ig: Immunoglobulin
ILAR: International League of Associations for Rheumatology
JIA: Juvenile idiopathic arthritis
MARAJIA: **M**ethotrexate **A**dvice and **R**ecommend**A**tions on **J**uvenile **I**diopathic **A**rthritis
MISS: MTX Intolerance Severity Score
MMR: Measles, mumps, rubella
MTX: Methotrexate
NSAIDs: Nonsteroidal anti-inflammatory drugs
PICO: Population – Intervention – Comparator – Outcome
PPD: Purified protein derivative
RF: Rheumatoid factor
TNF-α: Tumor necrosis factor-α
VZV: Varicella zoster virus
